# *Allium ducissae* (*A.* subgen. *Polyprason*, Amaryllidaceae) a New Species from the Central Apennines (Italy)

**DOI:** 10.3390/plants11030426

**Published:** 2022-02-04

**Authors:** Fabrizio Bartolucci, Marco Iocchi, Olga De Castro, Fabio Conti

**Affiliations:** 1 Scuola di Bioscienze e Medicina Veterinaria, Università di Camerino—Centro Ricerche Floristiche dell’Appennino, Parco Nazionale del Gran Sasso e Monti della Laga, San Colombo, Barisciano, 67021 L’Aquila, Italy; fabrizio.bartolucci@unicam.it (F.B.); fabio.conti@unicam.it (F.C.); 2 Viale Bruno Buozzi 59/C, 61032 Fano, Italy; marco.iocchi@gmail.com; 3 Dipartimento di Biologia, Orto Botanico, Università degli Studi di Napoli Federico II, 80139 Naples, Italy

**Keywords:** Abruzzo, *Allium strictum*, endemism, Lazio, Mediterranean flora, molecular phylogeny, nomenclature, nuclear DNA, plastid DNA, regional Park, taxonomy, typification, sect. *Daghestanica*, sect. *Falcatifolia*

## Abstract

In this paper, *Allium ducissae* (the LSID for the name *Allium ducissae* is: 77254606-1) is described as a new species based on morphological and molecular analyses, and its taxonomic relationships are discussed. It grows in crevices on calcareous rocks, rocky slopes and grassy ledges in the subalpine belt, within two regional protected areas in the Lazio and Abruzzo administrative regions (Central Apennines, Italy). Previously, these populations were attributed to *A. strictum*, a species described from Siberia, belonging to *A.* sect. *Reticulatobulbosa*. The new species is distinct from *A. strictum* in the morphology of vegetative and reproductive structures. Indeed, it is close to *A. palentinum*, an endemic species to Cantabrian Mountains (NW Spain). Both molecular and morphological data support the recognition of the *Allium* populations coming from the Central Apennines as a new species. *Allium ducissae* can be clearly distinguished from *A. palentinum* by longer and wider tepals, longer filaments, tooth of inner filament, flower pedicels, spathe appendage, and smaller seeds. Moreover, seed testa micro-sculptures revealed slight differences between *A. ducissae* and *A. palentinum*. Chromosome counts showed that *A. ducissae* is diploid with 2n = 16 chromosomes, as already known for *A. palentinum*. Molecular analyses support the affiliation of *A. ducissae* and *A. palentinum* to *A.* sect. *Falcatifolia*, contrary to what is known for the latter species, usually included in *A.* sect. *Daghestanica*. Finally, the IUCN assessment for the newly described species is proposed and briefly discussed.

## 1. Introduction

The genus *Allium* L. (Amaryllidaceae) is one of the richest and largest monocotyledonous genera, comprising about 1000 species [1,2]. It is distributed in temperate, semi-arid and arid regions of the Northern Hemisphere, with very rare exceptions [3,4]. The center of diversity of the genus extends from the Mediterranean basin to Central Asia [5]. Based on molecular phylogenetic studies, the genus *Allium* has been divided into 15 subgenera and 72 sections [4], which have been further classified into three evolutionary lineages. The classification of the genus is continuously revised and updated (e.g., [6,7,8]). With regards to Italy, *Allium* is represented in 78 taxa (species and subspecies), of which twenty-five are endemic and nine are alien species [9,10].

The present study aims to critically review the taxonomy and systematics of some peculiar *Allium* populations occurring on the mountains of the Central Apennines in the Lazio and Abruzzo administrative regions (Italy). These populations are currently attributed to *A. strictum* Schrad. [9,11,12,13,14,15], a species described from Siberia [16] belonging to *A.* subgen. *Reticulatobulbosa* (Kamelin) N.Friesen sect. *Reticulatobulbosa* Kamelin [4]. The latter section is characterized by sub-conical bulbs inserted above a short rhizome, with reticulated tunics and linear, flat leaves [4,17]. *Allium strictum* is a Eurasian species distributed in Europe, Russia, Kazakhstan, Kyrgyzstan, Mongolia and China [12,18,19]. Some authors (e.g., [13,20]) have highlighted the uncertain systematic position of the Apennines’ populations of *A. strictum*, suggesting further study. Based on a preliminary morphological analysis, these populations showed peculiar features, and turned out to be morphologically different from *A. strictum*, and therefore obviously cannot belong to the same species [21]. These populations are distinguished from *A. strictum* by coriaceous bulb tunics (vs. reticulate), leaves with smooth margins (vs. scabrous-denticulate margins), stamens that are long-exserted (vs. usually slightly longer than perianth segments), inner filaments with an acute tooth on each side (vs. rounded tooth), and conical stigma (vs. subcapitate to capitate stigma). Based on our preliminary observations, the Apennines populations belong to *A.* subgen. *Polyprason* Radić, and are close to *A. palentinum* Losa & P.Monts., an endemic species to the Cantabrian Mountains (NE Spain [22,23,24]). This latter species is classified in *A.* sect. *Daghestanica* (Tscholok.) N.Friesen [25], a section including the European species *A. ericetorum* Thore, *A. ochroleucum* Waldst. & Kit., *A. kermesinum* Rchb. [26], and *A. suaveolens* Jacq. These species are distributed from Carpathians across the Balkans and the Central Apennines to northern Spain and Portugal [9,24,27,28,29].

An extensive morphological and molecular investigation has been carried out on living specimens and herbarium material coming from the Central Apennines (Italy) and the Cantabrian Mountains, providing evidence about the species differentiation between *A. palentinum* and the Apennines’ populations. These results, and the disjunct and isolated geographical distribution of the populations occurring in the Central Apennines, allow us to refer to them as a species new to science, named *Allium ducissae*.

## 2. Materials and Methods

### 2.1. Plant Material and Morphological Analyses

This study is based mainly on field surveys, on an extensive analysis of relevant literature, and on the examination of herbarium specimens preserved at APP, BCN, MA, PI, and SALA (herbarium codes according to [30]) (Supplementary File S1). Morphological observations and measurements of 21 quantitative characters, considered as diagnostic in *Allium*, were analyzed on fresh material and dried herbarium specimens (Table 1). A total of 52 individuals of the new species (“DUC”) from three localities in the Central Apennines (Lazio: Mt. Morrone and Murolungo; Abruzzo: Mt. Rozza), and 45 of *A. palentinum* (“PAL”) from several localities in the Cantabrian Mountains (NE Spain), were analyzed. For each quantitative character, the Shapiro–Wilks normality test was first used to determine their distribution, then an independent sample *t*-test, after logarithmic transformation were carried out with SPSS ver. 25 software (IBM Corp., Armonk, NY, USA) [31]. Principal coordinate analysis (PCoA) was performed in PAST package ver. 4.04 software (Natural History Museum, Oslo, Norway) [32,33]. Some samples with missing data were not included in the principal coordinate analysis. Furthermore, the variability of the analyzed morphological characters was described by standard statistical parameters (mean, standard deviation, minimum, maximum, 10th and 90th percentiles). Boxplots were built through SPSS version 25. All morphological characters of dried material were observed with a Leica MZ16 stereoscopic microscope.

### 2.2. Chromosome Count

Chromosomes of the new species were counted from two individuals (on three metaphase plates) collected in the field on Mt. Morrone in loc. Fonte La Vena (July 2021) and then cultivated at the Floristic Research Centre of the Apennines (accession number 584/21). A voucher specimen has been deposited in the *Herbarium Apenninicum* (APP No. 66113). Squash preparations were made on ovules collected from living plants. Ovules were pretreated with a 0.4% colchicine solution for 4 h at room temperature and then fixed in Carnoy’s solution for 45 min. Then, they were hydrolyzed in 1 N HCl solution for 6 min at 60 ° C and stained with leucobasic fuchsine. Finally, they were squashed on clean glass slides with one drop of 45% acetic acid, before examination under a Leica DM750 light microscope for chromosome counting. 

### 2.3. Scanning Electron Microscopic Analyses

Seed testa micro-morphology on mature and dry material (five seeds per species) was analyzed. For the new species the seeds were collected from cultivated plants (from Mt. Rozza, Central Italy, Abruzzo) at the Botanical Garden of Floristic Research Centre of the Apennine, for *A. palentinum* were collected from herbarium specimen SALA barcode 136402 (loc. Valle de Valverde, Castiglia and León, Northern Spain,). Before the analysis with scanning electron microscope Zeiss Gemini SEM 500 at an accelerating voltage of 7 kV, dried seeds were attached to carbon-coated aluminium sample blocks. Terminology of the seed coat sculpture follows Barthlott [34], and Baasanmunkh et al. [35].

### 2.4. Molecular Analyses

Genomic DNA extraction. Four individuals from herbarium specimens housed in MA and APP herbaria (MA barcodes: MA532503, MA778505, MA515202, and SALA barcode: SALA136402; APP No. 66113, APP No. 66059, APP No. 66066, and APP No. 35345) were collected in different localities belonging to *A. ducissae* sp. nov. and *A. palentinum* and used for the phylogenetic analysis (Supplementary Table S1). Total genomic DNA from herbaria specimens was isolated by a modified CTAB 2X procedure [36] after the tissue pulverization using an UltraCool GeneReady Homogenizator (Life Real). DNA quality was checked with both spectrophotometer NanoReady Touch (Life Real) and 1% agarose electrophoresis with SafeView Nucleic Acid Stain (Applied Biological Materials, Vancouver, Canada) and visualized using the UVIdoc HD5 gel documentation system (UVITEC, Cambridge, U.K.). DNA concentration was estimated using a Qubit dsDNA HS Assay Kit with the Qubit 3 Fluorometer (Invitrogen, Thermo Fisher Scientific, Waltham, MA, USA).

PCR amplification and sequence analyses. The internal transcribed spacers (ITS; i.e., ITS1, ITS2 plus 5.8S gene) from nuclear DNA (nrDNA) and three intergenic spacers (IGS) (*trn*L^(UAA)^-*trn*F^(GAA)^, *trn*Q^(UUG)^-*rps*16, and *rp*L32-*trn*L^(UAG)^) from plastid DNA (cpDNA) were selected for molecular systematic analyses, being already employed in *Allium* phylogenies and taxonomy with special attention to *A.* sect. *Daghestanica* (e.g., [4,5,25,37,38,39,40,41]). 

ITS was amplified with a forward primer which anneals in the 3′region of the 18S (5′-GGA GAA GTC GTA ACA AGG TTT CCG-3′) as reported in Aceto et al. [42] and SN3 (reverse) by De Castro et al. [43]; ITS4 was used as an additional internal reverse for sequencing [44]. Plastidial markers were amplified using primers designed by Taberlet et al. [45] for *trn*L^(UAA)^-*trn*F^(GAA)^ IGS (c and f primers which amplified also the *trn*L^(UAA)^ intron), Shaw et al. [46] for *trn*Q^(UUG)^-rps16 (trnQ(UUG) and rpS16x1 primers) and *rp*L32-*trn*L^(UAG)^ IGS (rpL32-F and trnL(UAG) primers).

Amplification reaction used a volume of 20 μL, with 5–9 ng of DNA template, 0.25 μM of each primer, and Phire Plant Direct PCR Master Mix (Thermo Fisher Scientific) according to the manufacturer’s instructions. Annealing temperature (Ta) was equal to 55 °C for ITS and *trn*L^(UAA)^-*trn*F^(GAA)^ IGS and 50 °C for the other two plastid IGS markers.

The amplicons were purified using PEG8000 precipitation (PEG 15%, 2.5 M NaCl). Approximately 3–8 ng of purified template was sequenced in a final volume of 5 μL according to the instruction of the Bright Dye Terminator Cycle Sequencing Kit (MCLAB, San Francisco, CA, USA). The reactions were purified using the BigDye XTerminator Purification Kit (Applied Biosystems, Thermo Fisher Scientific, Waltham, MA, USA) and read using an automated sequencer (3130 Genetic Analyzer, Life Technologies, Thermo Fisher Scientific, Waltham, MA, USA). The sequences were analyzed using the AB DNA Sequencing Analysis ver. 5.2 software (Applied Biosystems, Thermo Fisher Scientific), edited in the Chromas lite ver. 2.6.6 software (Technelysium Pty Ltd., South Brisbane, Australia), assembled in the Chromas Pro ver. 2.1.8 software (Technelysium Pty Ltd., South Brisbane, Australia), aligned and analyzed using BioEdit ver. 7.2.5 software [47]. Sequences obtained in this study were deposited in GenBank under accession numbers OM030255-OM030262 (ITS), OM032824-OM032835 (*trn*Q^(UUG)^-rps16 and *trn*L^(UAA)^-*trn*F^(GAA)^ IGS), and OM055643-OM055646 (*rp*L32-*trn*L^(UAG)^ IGS) (Supplementary Table S1).

*Phylogenetic analyses*. After both a comparison of data in the literature (e.g., [25,39,40]) and our preliminary phylogenetic analyses, data sets from nrDNA and the combined cpDNA markers were analyzed separately. To know the systematic position of our new taxa, we selected from GenBank the *Allium* ITS accessions of almost the entire *A.* sect. *Daghestanica* (12 species for a total of 23 GenBank accessions) and some accessions representative of sect. *Falcatifolia* (3 species for a total of 10 GenBank accessions), sect. *Oreiprason* Hermann and sect. *Reticulobulbosa* (4 and 1 species, respectively, for a total of 10 GenBank accessions) (Supplementary Table S1). For the analysis of three plastid markers as a combined dataset, the accessions from GenBank were reduced in number to try to have the same specimen of the species used for the analysis of ITS, or at least that the plastid markers were always coming from the same specimen. Considering this and according to the ITS phylogeny results, 11 species (16 GenBank accessions) of *A.* sect. *Daghestanica* and 3 species (9 GenBank accessions) of *A.* sect. *Falcatifolia* were downloaded from GenBank (Supplementary Table S1). *Allium cyathophorum* Bureau & Franch. [*A.* subgen. *Cyathophora* (Fritsch) Fritsch] was used as an outgroup for both analyses using both nuclear and plastid markers. The sequence accessions, herbarium code, locality information and literature references are listed in Supplementary Table S1.

The phylogenetic relationships were assessed both with Bayesian (BI) and maximum likelihood (ML) inference on a dataset comprising 44 and 26 previously published ITS and plastid concatenated sequences, respectively, plus eight new specimens (four of both *A. ducissae* sp. nov. and *A. palentinum*). The most likely substitution models for nuclear and plastid markers were computed with jModeltest ver. 2.1.10 software [48]. The better model according to the Akaike information criterion (AIC) was GTR + G for ITS marker, TVM + G for *trn*Q^(UUG)^-*rps*16 IGS, and *rp*L32-*trn*L^(UAG)^ and TIM1 + G for *trn*L^(UAA)^-*trn*F^(GAA)^ IGS.

For both nuclear and plastid datasets, MrBayes ver. 3.2.6 software [49] was used for BI and two runs of four Markov chains (three hot, one cold) were performed for 15,000,000 generations, sampling every 1500 generations, and discarding the first 10% and 19% as burn-in (ITS and plastid markers, respectively). Convergence diagnostics were checked with Tracer ver. 1.7.1 software [50]. According to the cpDNA markers the models GTR + G (*trn*L^(UAA)^-*trn*F^(GAA)^ and *rp*L32-*trn*L^(UAG)^ IGS) and GTR + I + G (*trn*Q^(UUG)^-*rps*16) were used, being closer to those models previously calculated with jModeltest. Finally, an ML inference was performed on both datasets using RaxML-NG via its web server portal (https://raxml-ng.vital-it.ch/#/; [51]; accessed date, 26 November 2021). Bootstrap analyses were carried out with an automatic number of replicates with a bootstopping cut-off of 0.03.

## 3. Results

### 3.1. Taxonomic Treatment

**Figure 1:**
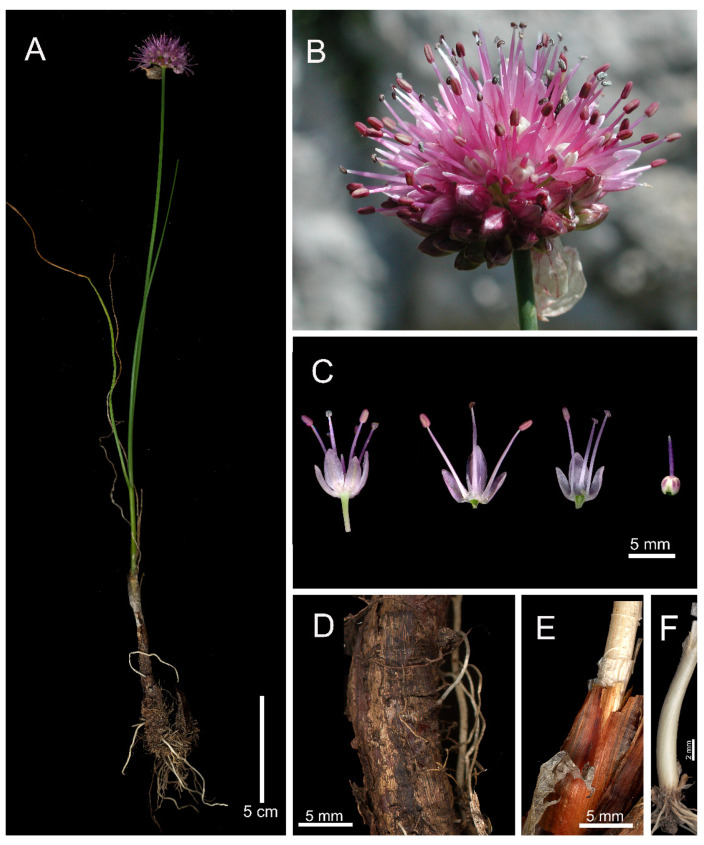
*Allium ducissae.* (**A**) Habit. (**B**) Inflorescence (Murolungo, Duchessa mountains, Abruzzo, Italy; photo by M. Iocchi). (**C**) Flowers, tepal and filament arrangement, pistil. (**D**) Outer bulb tunic. (**E**) Inner bulb tunic. (**F**) Bulb inserted on a short rhizome.

*Allium ducissae* Bartolucci, Iocchi & F.Conti, sp. nov. Figure 1.

—*Allium lineare* auct. fl. Ital. pro parte;

—*Allium strictum* auct. fl. Ital. pro parte.

**Figure 2:**
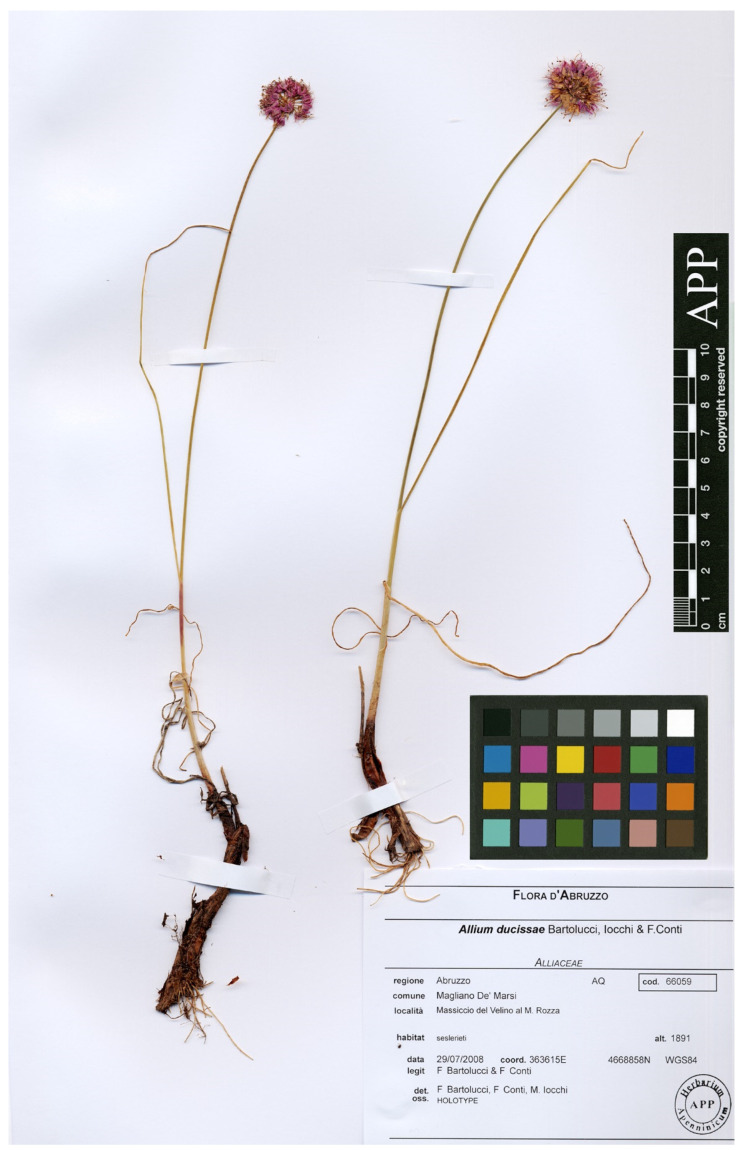
Holotype of *Allium ducissae* (APP No. 66059, reproduced with permission of the Herbarium, Centro Ricerche Floristiche dell’Appennino, Italy).

Type: Italy. Abruzzo, Massiccio del Velino al M. Rozza (WGS84: 4668858N, 363615E), seslerieti, 1891 m, 29 Jul 2008, *F. Bartolucci and F. Conti s.n.* (holotype: APP No. 66059, Figure 2; isotypes: APP Nos. 66060, 66061).

Diagnosis: *Allium ducissae* differs from *A. palentinum* by bulb (3.5) 5–6.68 (10) vs. (3) 6–10 (19) mm wide, stem (190) 302.5–448.75 (540) vs. (45) 157–260 (480) mm high, leaf smooth at margin vs. smooth or papillose; spathe appendage (0.62) 1.5–3.58 (5.5) vs. (0) 0.33–1.29 (2.7) mm long, flower pedicel (4) 5.5–6.9 (7.8) vs. (1.6) 2.75–4 (6.9) mm long, inner tepal (4) 5–5.5 (6) × (1.72) 2–2.29 (2.72) vs. (3.4) 4–4.9 (5.5) × (0.9) 1.6–2 (2.3) mm, outer tepal (3) 4–4.5 (5.1) × (1.4) 1.6–1.9 (2.1) vs. (3) 3.5–4.2 (4.6) × (1) 1.2–1.5 (1.9) mm, inner filament (6.3) 7.33–8.15 (8.9) vs. (4) 5.55–7 (9.2) mm long, outer filament (4) 6.5–7.98 (8.52) vs. (4) 5.55–6.7 (9.3) mm long, basal teeth of the inner filaments (0.1) 0.2–0.6 (1.3) vs. (0.02) 0.1–0.19 (1) mm long, seed (2.93) 3.23–3.57 (3.83) vs. (2.85) 3.41–4 (4.2) mm long.

Description: bulb almost cylindrical, (35) 50.5–70 (95.8) mm long and (3.5) 5–6.68 (10) mm wide, inserted on a short rhizome. Outer tunics coriaceous, dark brownish, the inner ones membranous, golden brown, shining. Stem (190) 302.5–448.75 (540) mm tall, terete, (0.8) 0.9–1.3 (1.8) mm in diameter, smooth, erect, covered for 1/3 (rarely 1/2) of its length by the leaf sheaths. Leaves (2) 3–4 (6), linear, flat, glabrous and smooth on margins, (1) 1.5–2.2 (3.5) mm wide. Spathe 1- or 2-valved, usually persistent, shorter than the inflorescence, with appendage (0.62) 1.5–3.58 (5.5) mm long. Inflorescence subglobose, (15) 16.88–23.36 (32) mm in diameter, many flowered (14–60), with smooth pedicels, subequal in length, (4) 5.5–6.9 (7.8) mm long, without bulbils. Perigon campanulate, pink, inner tepals longer than outer, elliptical, slightly eroded at margins and apex, (4) 5–5.5 (6) mm long and (1.72) 2–2.29 (2.72) mm wide, the outer ones ovate–oblong, (3) 4–4.5 (5.1) mm long and (1.4) 1.6–1.9 (2.1) mm wide. Stamens exserted, pink, the inner filaments (6.3) 7.33–8.15 (8.9) mm long with acute tooth on each side at base (rarely only one) (0.1) 0.2–0.6 (1.3) mm long, the outer ones, simple or with one tooth 0.1–0.2 mm mm long, (4) 6.5–7.98 (8.52) mm long. Anthers purple, elliptical, (0.7) 0.93–1.3 (1.6) mm long. Ovary subglobose, smooth, whitish with pink strips, (1.6) 1.8–2 (2.2) mm long and (1.7) 1.9–2 (2.1) mm wide, with large nectariferous pores, (0.4) 0.5–0.55 (0.6) × (0.4) 0.5–0.6 (0.65) mm. Style pink, (1.66) 3–5.52 (6.64) mm long, stigma conical. Capsule tri-valved, subglobose-obovate, greenish, (3.1) 3.5–4 (4.25) mm long and (3.3) 3.5–3.9 (4) mm wide, with evident nectariferous pores. Seeds elliptical-angular, (2.93) 3.23–3.57 (3.83) mm long.

**Figure 3:**
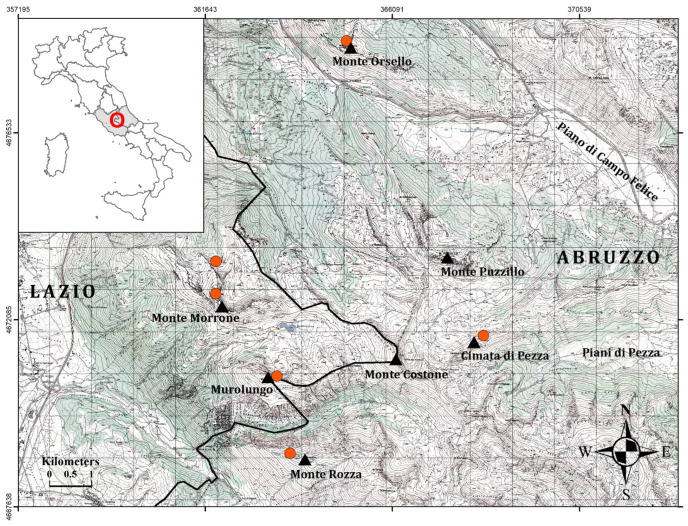
Map showing distribution of *Allium ducissae* in the Central Apennines, Lazio and Abruzzo, Italy. Latitude and longitude coordinates are expressed in meters using the WGS84 UTM 33N projected coordinate system.

**Figure 4:**
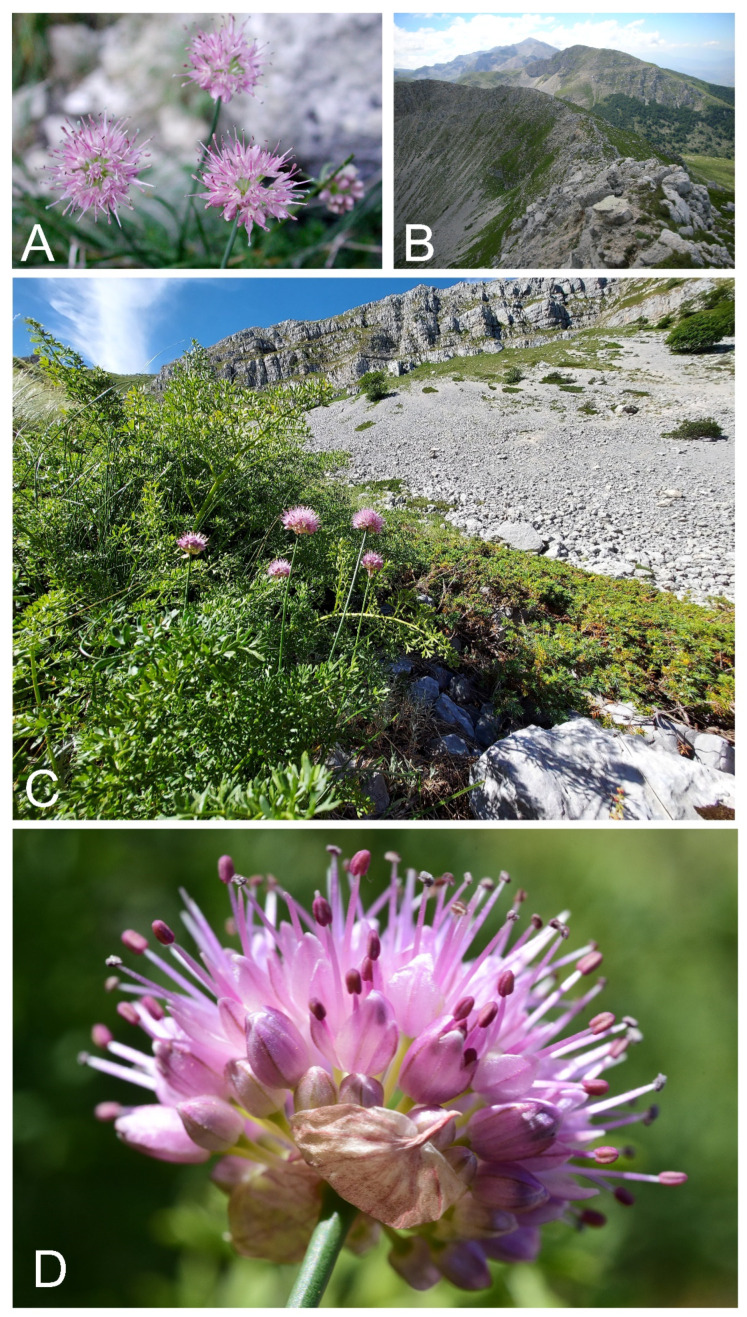
Habitat and flowering plants of *Allium ducissae.* (**A**) *A. ducissae* growing at the base of the calcareous wall of Murolungo (Duchessa mountains, Lazio, Italy; photo by M. Iocchi). (**B**) Top of Mt. Morrone in the foreground, in the background Murolungo and Mt. Velino (Lazio and Abruzzo, Italy; photo by M. Iocchi). (**C**) *A. ducissae* in loc. Fonte La Vena on Mt. Morrone (Duchessa mountains, Lazio, Italy; photo by F. Bartolucci). (**D**) Close up of *A. ducissae* growing at Fonte La Vena (Duchessa mountains, Lazio, Italy; photo by F. Conti).

Distribution and ecology: The narrow endemic *Allium ducissae* is distributed in the Central Apennines within the Velino massif at Mt. Rozza, Mt. Morrone and Murolungo (Duchessa mountains), Mt. Orsello and Cimata di Pezza (Lazio and Abruzzo administrative regions, Italy) (Figure 3). It is a microthermic taxon that grows in paucispecific primary habitat, such as crevices on calcareous rocks, rocky slopes, steppe grassland and grassy ledges, from 1800 up to 2130 m a.s.l (Figure 4), usually associated with *Sesleria juncifolia* Suffren subsp. *juncifolia*, *Carex kitaibeliana* Degen ex Bech., *Oreojuncus monanthos* (Jacq.) Záv.Drábk. & Kirschner, *Globularia meridionalis* (Podp.) O.Schwarz, *Helianthemum oelandicum* (L.) Dum.Cours. subsp. *incanum* (Willk.) G.López, *Sempervivum arachnoideum* L., *Lomelosia graminifolia* (L.) Greuter & Burdet subsp. *graminifolia* and *Grafia golaka* (Hacq.) Rchb. The vegetation types belong to the following syntaxa: *Seslerion apenninae* Bruno and Furnari 1966, *Potentillion caulescentis* Br.-Bl. in Br.-Bl. and Jenny 1926.

Phenology: flowering from July to August, fruiting in September.

Etimology: *Allium ducissae* is named after the Duchessa mountains (in Latin, Montis Ducissae) where the species was discovered for the first time by Bruno Petriccione (under the name *A. lineare*, [11]).

**Figure 5:**
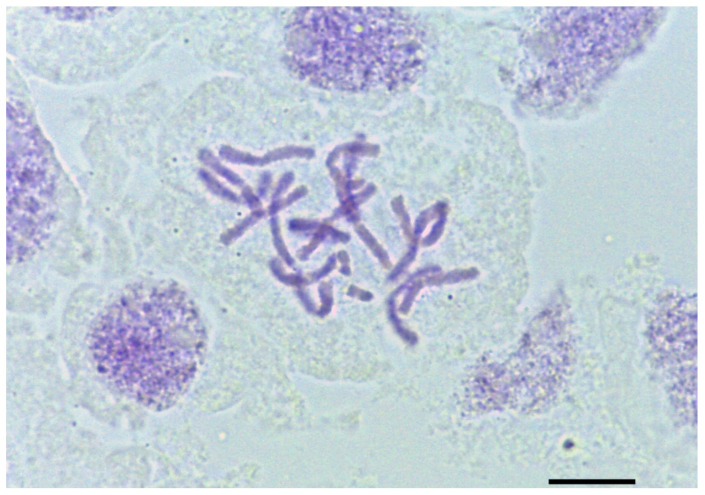
Mitotic metaphase plate of *Allium ducissae* 2n = 16 + 2B. Image obtained with a Leica DM750 light microscope (original magnification: 1000×). Scale bar: 10 μm.

Chromosome number: a single population from “Mt. Morrone in loc. Fonte La Vena” (Lazio, Rieti province) of *A. ducissae* resulted in diploid with 2n = 16 + 2B chromosomes (Figure 5). This chromosome count agrees with previous counts made for *A. palentinum* [52].

**Figure 6:**
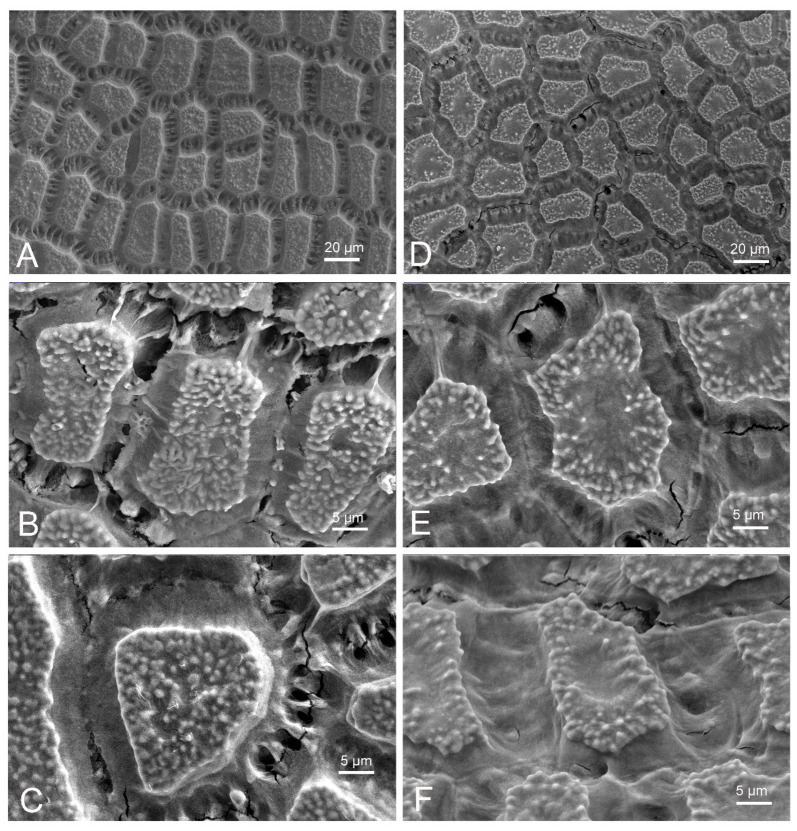
Scanning electron micrographs of seed testa sculptures. (**A**–**C**) *Allium ducissae*. (**D**–**F**) *A. palentinum*.

Seed micromorphology: seeds of *Allium ducissae* showed irregularly polygonal testa cells with a broad, depressed and coarsely striated intercellular region. The anticlinal walls appeared depressed, straight to curved. The periclinal walls are flat, and densely granulate (Figure 6A–C). The seeds of *A. palentinum* showed irregularly polygonal testa cells, with a broad, depressed intercellular region with obscure striation. The anticlinal walls appeared depressed, straight to curved. The periclinal walls are irregularly granulose (mainly on the margin), with a concave center (Figure 6D–F).

Conservation status: *Allium ducissae* occurred in the NATURA 2000 network within the Sites of Community Interest “IT6020020 Monti della Duchessa” and “IT7110206 Monte Sirente e Monte Velino” in the Regional Natural Reserve “Montagne della Duchessa” (Lazio) and the Regional Park “Sirente Velino” (Abruzzo), respectively. Only the subpopulation of Mt. Orsello is not included in a protected area. The extent of occurrence (EOO) is 32.91 km^2^, calculated with minimum convex hull polygon in QGIS, and the area of occupancy (AOO) is 24 km^2^, calculated with a 2×2 km cell fixed grid. The main estimated threats include: global warming, which favors the arrival of more thermophilous competitive species; poor level of reproduction and regeneration, that leads to a decreasing population trend; restricted range, which causes a greater risk of extinction in the event of disease or pest attacks. The taxon occurs in one location and five subpopulations. The population is not declining and there are no extreme fluctuations. According to IUCN criteria [53], we propose to include *A. ducissae* in the following category: Near Threatened (NT).

### 3.2. Morphological Analyses

**Figure 7:**
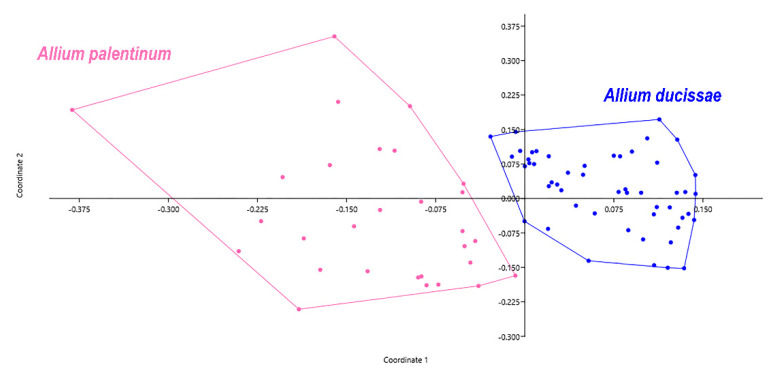
Principal coordinates analysis (PCoA) based on the distance matrix of Bray–Curtis dissimilarity. Scatter plot of first two principal coordinate axes based on 21 morphological characters and 80 specimens.

**Figure 8:**
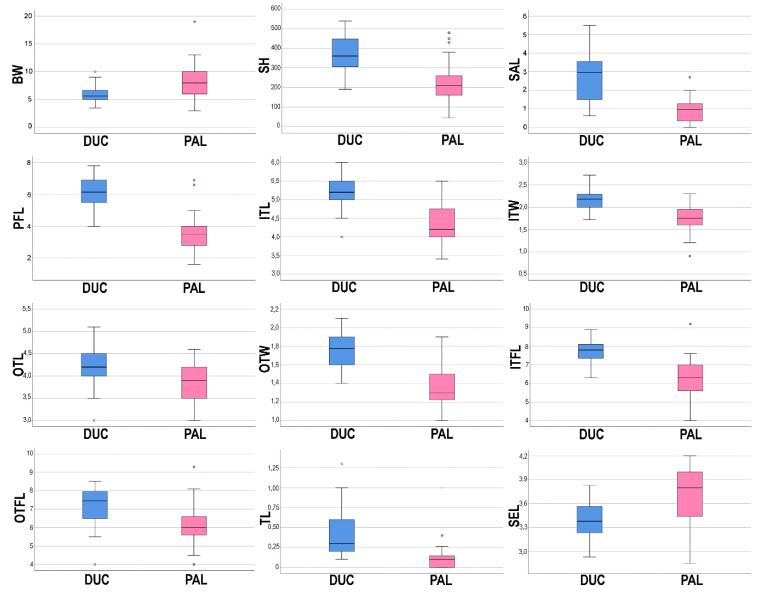
Boxplots expressing morphological variation between *Allium ducissae* (DUC) and *A. palentinum* (PAL): bulb width (BW), stem height (SH), spathe appendage length (SAL), pedicel flower length (PFL), inner tepal length (ITL), inner tepal width (ITW), outer tepal length (OTL), outer tepal width (OTW), inner filament length (ITFL), outer filament length (OTFL), tooth of inner filament length (IFTL), seed length (SEL). Outlined central box depicts middle 50% of data, extending from 25th and 75th percentiles, and horizontal bar is the median. Ends of vertical lines (or “whiskers”) indicate minimum and maximum data values, unless outliers are present, in which case whiskers extend to a maximum of 1.5 times inter-quartile range. Circles indicate outliers.

**Figure 9:**
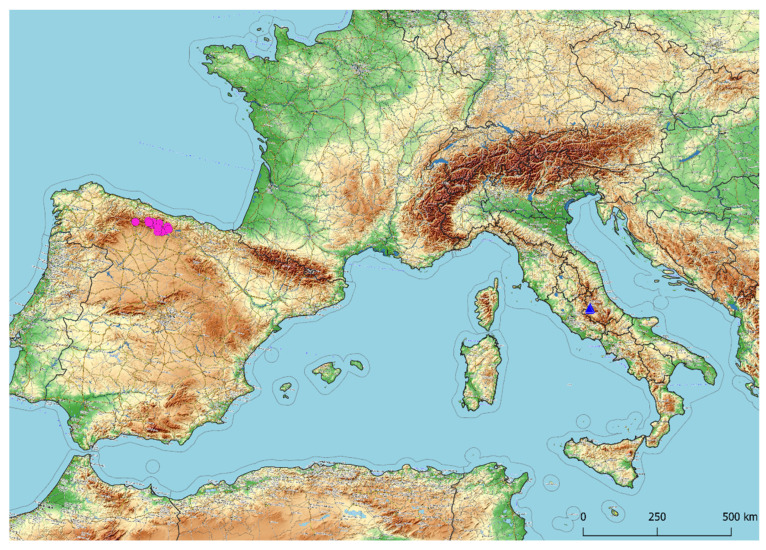
Distribution map of *Allium ducissae* (blue triangles), and *A. palentinum* (pink circles) according to the herbarium material examined. The map was used under a CC BY-SA copyright from OpenStreetMap contributors.

The principal coordinate analysis (PCoA, Figure 7) shows on the first two axes a clear distinction between *A. ducissae* and *A. palentinum,* and no overlapping areas among individuals were found. The most relevant morphological characters differentiating the two species are summarized in Table 2 and shown in Figure 8. The states of twelve characters (BW, SH, SAL, PFL, ITL, ITW, OTL, OTW, ITFL, OTFL, IFTL and SEL) show significant differences between the two species (*p* < 0.01). The geographic distribution of the herbarium specimens examined (Supplementary File S1) is shown in Figure 9. The map was created using the free and open source QGIS ver. 3.16.4. software [54].

### 3.3. Molecular Analyses

The alignment of 52 ITS sequences (Supplementary Table S1) resulted in a matrix of 658 characters, 258 of which were variable and 205 parsimony informative sites (Supplementary File S2). The concatenated matrix of three cpIGS markers (*trn*Q^(UUG)^-*rps*16, *trn*L^(UAA)^-*trn*F^(GAA)^, and *rp*L32-*trn*L^(UAG)^) of the 34 taxa (Supplementary Table S1) generated a matrix of 1764 characters, with 241 variable sites and 155 parsimony informative sites (Supplementary File S3).

**Figure 10:**
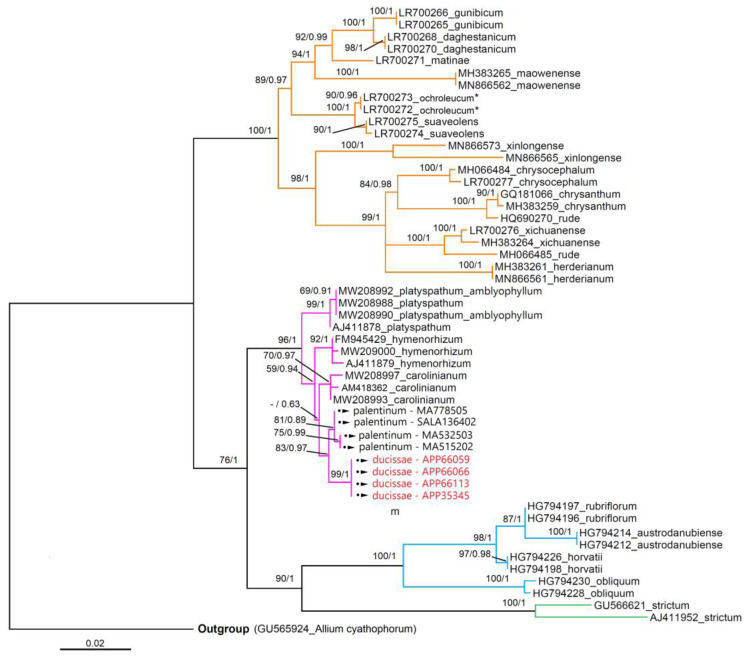
Maximum likelihood (ML) phylogram of newly generated and previously published ITS sequences of *Allium* (GenBank code + specific epithet). The newly generated sequences have symbol (•►) and in red the samples of *A. ducissae* sp. nov. Accessions belonging to *A.* sect. *Daghestanica* are shown with orange branches, sect. *Falcatifolia* in pink, sect. *Oreiprason* and *Reticulobulbosa* in blue and green, respectively. ML bootstrap values followed by Bayesian posterior probabilities are shown above the branches (BS/PP values > 50%). *, species reported in Freisen et al. (2020) as *A. ericetorum* Thore. For information of the samples, see Supplementary Table S1 and for ITS matrix see Supplementary File S2.

**Figure 11:**
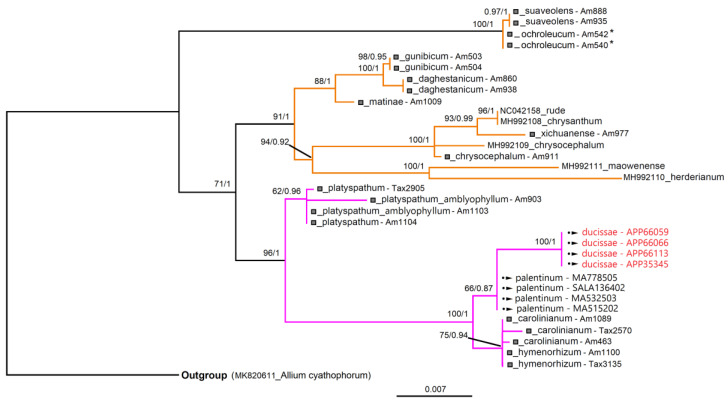
Maximum likelihood (ML) phylogram of newly generated and previously published plastid sequences of Allium (*trn*Q^(UUG)^-*rps*16, *trn*L^(UAA)^-*trn*F^(GAA)^, and *rp*L32-*trn*L^(UAG)^ IGS) (GenBank code or symbol (□) + specific epithet). The symbol (□) indicates that the sample has three GenBank codes corresponding to the analyzed markers. A single Genbank code corresponds to the plastid genome of the specimen. The newly generated sequences have symbol (•►) and in red the samples of *A. ducissae* sp. nov. Accessions belonging to *A.* sect. *Daghestanica* are shown with orange branches and sect. *Falcatifolia* in pink. ML bootstrap values followed by Bayesian posterior probabilities are shown above the branches (BS/PP values > 50%). *, species reported in Freisen et al. [25] as *A. ericetorum* Thore. For information of the samples see Supplementary Table S1 and for concatenated cpIGS matrix see Supplementary File S3.

The phylogenetic trees derived from BI and ML analyses were topologically identical and the ML trees are only shown for both molecular markers (nrDNA and cpDNA) (Figure 10 and Figure 11). The topology trees obtained were congruent with the literature data. (e.g., [25,39]) The posterior probabilities (PP) and bootstrap support (BS) showed high values in both analyses (Figure 10 and Figure 11). 

According to the ITS phylogenetic analysis (Figure 10), the accessions of *A. ducissae* sp. nov. and *A. palentinum* fit into a well-defined clade with other taxa belonging to *A.* sect. *Falcatifolia* N.Friesen; considering the tree topology, *A. palentinum* is close to *A. ducissae*. This datum was also confirmed with plastid data, as shown in Figure 11. 

## 4. Discussion

Morphological and molecular analyses provide evidence that *A. ducissae* should be regarded as a new species, endemic to Abruzzo and Lazio (Central Apennines, Italy). The populations of *A. ducissae* were previously referred to as *A. strictum* [9,11,12,13,14,15,20], a species morphologically different by having reticulate bulb tunics, leaves with a scabrous-denticulate margin, stamens usually slightly longer than perianth segments, inner filaments with a rounded tooth at the base, and subcapitate to capitate stigma [21]. Consequently, the occurrence of *A. strictum* from peninsular Italy should be excluded. This latter species is very rare in Italy, where it occurs only in the Alps, representing the western limit of its distribution area (e.g., [9,12,19,29]).

*Allium ducissae* is similar to *A. palentinum*, an endemic species of the Cantabrian Mountains (NW Spain), but it can be distinguished by several morphological characters, as shown in Table 1. It also showed slight differences in seed testa microsculptures, with densely granulated periclinal walls with a flat center (vs. mainly granulated at the margin with a concave center) and anticlinal walls with a coarsely striated intercellular region (vs. obscure striation).

Based on molecular analyses, both *A. ducissae* and *A. palentinum* belong to *A.* sect. *Falcatifolia*, contrary to what is known for the latter species, usually included in *A.* sect. *Daghestanica* [25]. Moreover, our results of seed testa micromorphology agreed with the findings for other species belonging to *A.* sect. *Falcatifolia* [35,55,56]. This latter section was originally described by Friesen [4] including only *A. carolinianum* DC. ex Redouté and *A. platyspathum* Schrenk; recently, however, this was newly circumscribed with the inclusion of several species [37,57,58]. *Allium ducissae* and *A. palentinum* are the westernmost representatives of this section, and the only ones present in Europe.

*Allium ducissae* joins the flora of the Central Apennines, an area with high plant species diversity, where there are the protected areas with the highest number of taxa in Europe and the Mediterranean Basin [59,60,61]. The Central Apennines are also known for being a large contingent of endemic taxa [62,63,64,65,66], some of which have recently been described or re-evaluated as, for example, *Anthyllis apennina* F.Conti & Bartolucci [67], *Corydalis densiflora* C.Presl subsp. *apennina* F.Conti, Bartolucci & Uzunov [68], *Crepis magellensis* F.Conti & Uzunov [69], *Gagea tisoniana* Peruzzi, Bartolucci, Frignani & Minut. [70], *Genista pulchella* Vis. subsp. *aquilana* F.Conti & Manzi [71], *Lathyrus apenninus* F.Conti [72], *Oxytropis ocrensis* F.Conti & Bartolucci [73], *Pinguicula vallis-regiae* F.Conti & Peruzzi, *P. vulgaris* subsp. *vestina* F.Conti & Peruzzi, *P. vulgaris* subsp. *ernica* Peruzzi & F.Conti, *P. vulgaris* subsp. *anzalonei* Peruzzi & F. Conti [74], *Poa magellensis* F.Conti & Bartolucci [75], *Senecio apenninus* Tausch [76], *Ranunculus giordanoi* F.Conti & Bartolucci [77], *R. bariscianus* Dunkel, *R. multidens* Dunkel, *R. pedrottii* Spinosi ex Dunkel [78], and *Sedum aquilanum* L.Gallo & F.Conti [79]. Endemics are the most vulnerable component of a flora, and many of the mentioned endemic taxa might become endangered or extinct due to their restricted area of distribution and strict ecological requirements. Systematic and taxonomic studies, and the phytogeographic analysis of endemic taxa are essential for setting conservation priorities and for driving in situ conservation measures and ex situ conservation activities [66,80,81].

The actual range of *A. ducissae* is restricted to the Velino massif, a territory in the Central Apennines with a distinctly continental climate. All the existing populations occur in paucispecific primary habitats, preferring northern exposures or other spots with long persistence of snow cover (microthermic conditions). The ecological features and the restricted distribution area could support the hypothesis that *A. ducissae* is a species differentiated in the Quaternary due to the fragmentation of the range of a widespread ancestral Tertiary taxon.

## Figures and Tables

**Table 1:** Morphological characters employed in the morphometric analyses.

	Abbreviations	Characters
1	BL	Bulb length (mm)
2	BW	Bulb width (mm)
3	SH	Stem height (mm)
4	SW	Stem width under the inflorescence (mm)
5	LW	Leaf max width (mm)
6	SAL	Spathe appendage length (mm)
7	ID	Inflorescence diameter (mm)
8	PFL	Pedicel flower length (mm)
9	ITL	Inner tepal length (mm)
10	ITW	Inner tepal width (mm)
11	OTL	Outer tepal length (mm)
12	OTW	Outer tepal width (mm)
13	ITFL	Inner filament length (mm)
14	OTFL	Outer filament length (mm)
15	IFTL	Tooth of inner filament length (mm)
16	AL	Anther length (mm)
17	OL	Ovary length (mm)
18	SL	Style length (mm)
19	SEL	Seed length (mm)
20	NL	Numbers of leaves
21	NF	Numbers of flowers

**Table 2:** Comparisons of morphological characters between *Allium ducissae* and *A. palentinum*. Quantitative continuous characters are expressed in mm and are reported as mean ± standard deviation and 25–75 percentiles (extreme values in brackets). For quantitative discrete cardinal characters, 25–75 percentiles are given (extreme values in brackets). Significantly different character states are shown in bold (*p* < 0.01).

	Character	*Allium ducissae*		*Allium palentinum*	
1	BL	62.1 ± 14.5	(35)50.5–70(95.8)	57.3 ± 14.7	(33)45–69.1(86.5)
2	BW	**5.93 ± 1.45**	**(3.5)5–6.68(10)**	**8.44 ± 2.97**	**(3)6–10(19)**
3	SH	**364.81 ± 86.67**	**(190)302.5–448.75(540)**	**226.73 ± 104.8**	**(45)157–260(480)**
4	SW	1.13 ± 0.27	(0.8)0.9–1.3(1.8)	1.29 ± 0.33	(0.7)1–1.6(2)
5	LW	1.95 ± 0.5	(1)1.5–2.2(3.5)	2.16 ± 0.78	(1.1)1.5–2.8(4.6)
6	SAL	**2.72 ± 1.31**	**(0.62)1.5–3.58(5.5)**	**0.88 ± 0.69**	**(0)0.33–1.29(2.7)**
7	ID	20.98 ± 4.5	(15)16.88–23.36(32)	18.23 ± 3.73	(13)15–20(27)
8	PFL	**6.15 ± 0.83**	**(4)5.5–6.9(7.8)**	**3.51 ± 1.11**	**(1.6)2.75–4(6.9)**
9	ITL	**5.25 ± 0.45**	**(4)5–5.5(6)**	**4.34 ± 0.52**	**(3.4)4–4.9(5.5)**
10	ITW	**2.16 ± 0.24**	**(1.72)2–2.29(2.72)**	**1.74 ± 0.3**	**(0.9)1.6–2(2.3)**
11	OTL	**4.25 ± 0.42**	**(3)4–4.5(5.1)**	**3.84 ± 0.42**	**(3)3.5–4.2(4.6)**
12	OTW	**1.73 ± 0.17**	**(1.4)1.6–1.9(2.1)**	**1.37 ± 0.23**	**(1)1.2–1.5(1.9)**
13	ITFL	**7.77 ± 0.59**	**(6.3)7.33–8.15(8.9)**	**6.33 ± 1.03**	**(4)5.55–7(9.2)**
14	OTFL	**7.15 ± 0.99**	**(4)6.5–7.98(8.52)**	**6.12 ± 1.13**	**(4)5.55–6.7(9.3)**
15	IFTL	**0.41 ± 0.29**	**(0.1)0.2–0.6(1.3)**	**0.18 ± 0.04**	**(0.02)0.1–0.19(1)**
16	AL	1.15 ± 0.25	(0.7)0.93–1.3(1.6)	1.13 ± 0.22	(0.8)1–1.3(1.5)
17	OL	1.92 ± 0.13	(1.6)1.8–2(2.2)	1.83 ± 0.3	(1.2)1.63–2(2.5)
18	SL	4.28 ± 1.44	(1.66)3–5.52(6.64)	4.01 ± 1.19	(2)3–5(6.6)
19	SEL	**3.39 ± 0.2**	**(2.93)3.23–3.57(3.83)**	**3.68 ± 0.38**	**(2.85)3.41–4(4.2)**
20	NL		(2)3–4(6)		(2)3–4(5)
21	NF		(14)22.25–36.5(60)		(6)14–30(60)

## Data Availability

The datasets generated in the current study are available from the corresponding author on reasonable request.

## Supplementary Materials

The following are available online at https://www.mdpi.com/article/10.3390/plants11030426/s1. File S1: List of the herbarium specimens examined. Table S1. List of *Allium* taxa used for the phylogenetic analyses (taxon, herbarium code, origin, GenBank accession, and reference). File S2: Alignment of 52 ITS sequences used for the BI and ML phylogenetic inferences. File S3: Alignment of 34 concatenated cpIGS sequences (*trn*Q^(UUG)^-*rps*16, *trn*L^(UAA)^-*trn*F^(GAA)^, and *rp*L32-*trn*L^(UAG)^) used for the BI and ML phylogenetic inferences.
