# Supplementary material for: Allium ducissae (A. subgen. Polyprason, Amaryllidaceae) a New Species from the Central Apennines (Italy)
Source: Plants (Basel). 2022 Feb 4;11(3):426. doi: 10.3390/plants11030426 (PMC8839878; doi:10.3390/plants11030426)
Supplement: Supplementary file 1 [file plants-11-00426-s001.zip › File S1.pdf]

List of the specimens examined

Allium ducissae Bartolucci, Iocchi & F.Conti (paratypes)

Italy. LAZIO: Grotta dell'Oro, Murolungo (Borgorose), 01 Aug 2005, *D. Valfrè s.n.* (APP No. 35345); Murolungo, versante N gruppo del Velino, AQ, 1900 m, 16 Aug 1991, *B. Petriccione s.n.* (PI No. 037791, under the name *Allium lineare* L.); Murolungo (m 1900), gruppo del Velino, AQ, arbusteti a *Juniperus nana*, esp. N, 12-16 Aug 1991, *B. Petriccione s.n.* (PI No. 037792, under the name *Allium lineare* L.); Montagne della Duchessa, Murolungo nei pressi della Grotta dell'Oro, ghiaioni e pascoli, 2000 m, 15 Jul 2008, *F. Bartolucci, M. Iocchi, L. Carotenuto & M. Cutini s.n.* (APP No. 66062); Montagne della Duchessa, Monte Morrone, nicchia tra le rupi calcaree, 16 Aug 2006, *M. Iocchi* (APP No. 62735); Montagne della Duchessa (Borgorose, Rieti), versante nord-orientale di Monte Morrone, cenge esposte a nord-est, 2127 m, 29 Jul 2008, *M. Iocchi & F. Bartolucci s.n.* (APP Nos. 66063, 66064, 66065); Monte Morrone, loc. Fonte La Vena (Borgorose, Rieti), ghiaioni calcarei, 1811 m, 26 Jul 2021, *Bartolucci F., Conti F.* (APP No. 66113); M. Duchessa (Borgorose, Rieti), alla base di fonte La Vena, tra grossi massi alla base del ghiaione, 1820 m, 15 Jul 2010, *M. Iocchi & D. Valfré s.n.* (URT); ABRUZZO: Val di Teve presso il rifugio Sevice (Magliano De' Marsi), 1850 m, 14 Sep 2001, *D. Valfrè s.n.* (APP No. 40591); Monte Orsello, macereto, 1900 m, 9 Jul 2006, *R. Soldati s.n.* (APP No. 66066); Massiccio del Velino al M. Rozza (WGS84: 4668858N, 363615E), seslerieti, 1891 m, 29 Jul 2008, *F. Bartolucci & F. Conti s.n.* (holotype: APP No. 66059, isotypes: APP Nos. 66060, 66061);

Allium palentinum Losa & P.Monts.: Montes leoneses, Riaño. Pico Yordas; Coriscao; Pico del Fraile en Sierra del Brezo, s.d., [*T.M. Losa? s.n.*] (BCN No. 15759, two sheets); Solana del Yordas. Riaño, 1750 m, 1 Aug 1950, [*P. Montserrat? s.n.*] (BCN No. 28701, two sheets); Pico Coriscao, 2220-2270 m, 4 Aug 1953, [*P. Montserrat? s.n.*] (BCN No. 28702); Umbría cumbre Pico del Fraile, 2010 m, 13 Aug 1953, [*P. Montserrat? s.n.*] (BCN No. 28703); Mampodre (León), Cervunal de Valverde en […], 2000 m, 5 Aug 1952, *T.M. Losa & P. Montserrat s.n.* (BCN No. 5072), Pico del Convento, Mampodre (León) […] de la parte septentrional, 1900 m, 5 Aug 1952, *T.M. Losa & P. Montserrat s.n.* (BCN No. 5075); Cumbre de Peña Redonda, ladera N. […], 23 Jul 1949, *T.M. Losa & P. Montserrat s.n.* (BCN barcode BCN2893); Leon: Puerto de las Señales, 16 Aug 1978, *E.Rico s.n.* (MA barcode MA219541); *ibidem*, 26 Jul 1979, *Casaseca et al. s.n.* (MA barcode MA223529); Maraña (Leon): Mamprode, laderas

pedregosas de la umbría a calizas (33T UN2266) , 1600-1900 m, *B. Fz. De Betoño & P. Urrutia 1481-86* (MA barcode MA365997); […] N del Coriscao (33T UN 5373), 1900 m, 14 Aug 1988, […] (MA barcode MA465460); Velilla de Río Carrión: Sierra del Brezo (30TUN 5544), roquedos de la umbría y laderas pedregosas de fuerte pe6 ndiente de la Peña del Fraile, calizas, 1880-2000 m, 13 Aug 1989, *B. Fz. De Betoño & J.A. Alejandre 1233/89* (MA barcode MA484071); Cervera de Pisuerga: Sierra del Brezo, Peña Redonda, gleras y laderas de la umbría, calizas, 1460-1620, 5 Aug 1989, *B. Fz. De Betoño & J.A. Alejandre 1079/89* (MA barcode MA484072); Pico Espigüete, Velilla del Río Carrión, Palencia, roquedos e pedregosas en la base de la cara N, UN5457, 1600 m, *V.J. Arán et al.* (MA barcode MA515202); Velilla del Río Carrión, Espigüete, roquedos e pedregosas de la umbría, en el circo N, en un ambiente general fuertemente carstificado (30TUN5356), calizas, 2020 m, 12 Aug 1990, *B. Fz. De Betoño & J.A. Alejandre 410/90* (MA barcode MA532503, SALA No. 58328); Palencia, Peña Labra, 33TUN86, 1700 m, 28 Jul 1984, *C. Aedo s.n.* (MA barcode MA599624); Sel de la Fuente, Valdecebollas, Palencia, roquedo calizo umbrío, 1840 m, 9 Aug 1987, *Moreno s.n.* (MA barcodes MA682626, MA-02-00682626); León, Maraña, valle de Valverde, 43°01′44′′N 05°11′44′′E, roquedo calizo, 1860 m, 13 Aug 2005, *C. Aedo 12268* (MA barcode MA731314, SALA barcode 136402); León, Viadangos de Arbas, valle de Tereina, pie de cinglo orient. N., 30TTN7559, 1350-1400 m, 6 Jul 2001, *J. Molero s.n.* (MA barcode MA738797); Palencia, ladera N del Espigüete, 42°56′34′′N 04°48′27′′W, datum WGS84, repisas de roquedo calizas, 2050 m, 8 Aug 2008, *C. Aedo 15873* (MA barcodes MA778505, MA-02-778505, MA-03-778505); Puerto de las Señales (León), 26 Aug 1968, *E Rico s.n.* (SALA No. 19259); *ibidem*, 26 Jul 1979, *Casaseca et al. s.n.* (SALA No. 22276); Picos de Europa, Aliva, 14 Aug 1980, *E Rico s.n.* (SALA No. 26773); León, Pico Lago, 30TUN1871, pastizal calizo, 1845 m, 30 Jul 1996, *C. Lence s.n.* (MA barcode MA-01-00925958); Asturias, Somiedo, Murias Longas (UTM: 29TQH36), gleres de cantos pequeños y móviles, 1600 m, 24 Aug 1885, *I. Aizpure s.n.* (MA barcode MA769131); Asturias, Peña Ubiña, 2100 m, 20 Aug 1974, *S. Castroviejo s.n.* (MA barcode MA433702); canal de la Jenduda, pr. Aliva, Pico de Europa, 22 Aug 1982, *C. Aedo s.n.* (MA barcode MA465567).
