# Supplementary material for: Allium ducissae (A. subgen. Polyprason, Amaryllidaceae) a New Species from the Central Apennines (Italy)
Source: Plants (Basel). 2022 Feb 4;11(3):426. doi: 10.3390/plants11030426 (PMC8839878; doi:10.3390/plants11030426)
Supplement: Supplementary file 1 [file plants-11-00426-s001.zip › Table S1.pdf]

Article: *Allium ducissae* (*A.* subgen. *Polyprason*, Amaryllidaceae) a new species from central Apennines (Italy)

Authors: Fabrizio Bartolucci, Marco Iocchi, Olga De Castro and Fabio Conti

Supplementary File.

Table S1. List of *Allium* taxa used for the phylogenetic analyses (taxon, herbarium code, origin, GenBank accession, and reference).

**Taxon**	**Code**	**Origin**	**GenBank accession**				
			**ITS**	***trn*Q$^{(UUG)}$-*rps*16**	***trn*L$^{(UAA)}$-*trn*F$^{(GAA)}$**	***rp*L32-*trn*L$^{(UAG)}$**	**Ref.**
Allium austrodanubiense N.Friesen & Seregin	GL-107	Bulgaria: Black Sea Coast, Kaliakra Reserve	HG794212	n.d.	n.d.	n.d.	1
A. austrodanubiense	GL-109	Bulgaria: Konjavsko Mt, Smudertsi	HG794214	n.d.	n.d.	n.d.	1
A. carolinianum DC.	Tax2570	Tajikistan: Anzob Pass	AM418362	LR700292	LR700307	LR700264	2
A. carolinianum	Am463	Mongolia: Dzungarian Gobi, Baytag Bogd	MW208993	MW201154	n.d.	MW201106	2
A. carolinianum	Am1089	Kazakhstan: Koyandytau, Tamschi	MW208997	MW201155	n.d.	MW201105	2
A. chrysanthum Regel	n.d.	China	MH383259	n.d.	n.d.	n.d.	3
A. chrysanthum	n.d.	China: Huzhu, Xining	GQ181066	n.d.	n.d.	n.d.	4
A. chrysanthum	n.d.	n.d.	n.d.	MH992108	MH992108	MH992108	5
A. chrysocephalum Regel	Am911	China: Gansu	LR700277	LR700290	LR700305	LR700262	6
A. chrysocephalum	n.d.	n.d.	MH066484	n.d.	n.d.	n.d.	7
A. chrysocephalum	n.d.	n.d.	n.d.	MH992109	MH992109	MH992109	5
A. cyathophorum Bureau & Franch.	n.d.	n.d.	GU565924	n.d.	n.d.	n.d.	8
A. cyathophorum	n.d.	China: Mangkang, Tibet	n.d.	MK820611	MK820611	MK820611	9
A. daghestanicum Grossh.	Am938	Russia: Daghestan, Danuch	LR700270	LR700283	LR700298	LR700255	6

A. daghestanicum	Am860	Russia: Daghestan, Shamilsky	LR700268	LR700281	LR700296	LR700253	6
A. ducissae Bartolucci, Iocchi & F.Conti	APP66113	Italy: Morrore Mt.	OM030255	OM032824	OM032832	OM055643	t.s.
A. ducissae	APP66059	Italy: Velino Mt.	OM030256	OM032825	OM032833	OM055644	t.s.
A. ducissae	APP66066	Italy: Orsello Mt.	OM030257	OM032826	OM032834	n.d.	t.s.
A. ducissae	APP35345	Italy: Murolungo	OM030258	OM032827	OM032835	n.d.	t.s.
A. gunibicum Miscz. ex Grossh.	Am503	Russia: Daghestan, Avgali	LR700265	LR700278	LR700293	LR700250	6
A. gunibicum	Am504	Russia: Daghestan, Verchnij Gunib	LR700266	LR700279	LR700294	LR700251	6
A. herderianum Regel	0701i		MH383261	n.d.	n.d.	n.d.	3
A. herderianum	n.d.	China: LianHuaShan, KangLe, GanSu	MN866561	n.d.	n.d.	n.d.	10
A. herderianum	n.d.	China: Kangle, Gansu	n.d.	MH992110	MH992110	MH992110	5
A. horvatii Lovric	AM-433	Italy: Monte Autore	HG794226	n.d.	n.d.	n.d.	1
A. horvatii	GL-89	Montenegro: Orjen Mt.	HG794198	MW201148	HG794050	HG794120	1
A. hymenorhizum Ledeb.	Tax 3135	Tajikistan: Saravshan	AJ411879	LR700291	LR700306	LR700263	6
A. hymenorhizum	Am1100	Kazakhstan: Region Ile Alatau	MW209000	MW201158	n.d.	MW201109	2
A. hymenorhizum	n.d.	Kyrgyzstan: Alaiskij Chrebet	FM945429	n.d.	n.d.	n.d.	11
A. maowenense J.M.Xu	n.d.	China: ShuiXiCun, MaoXian, SiChuan	MN866562	n.d.	n.d.	n.d.	10
A. maowenense	2601i	n.d.	MH383265	n.d.	n.d.	n.d.	3
A. maowenense	n.d.	China: Mao County, Sichuan	----	MH992111	MH992111	MH992111	5
A. matinae N.Friesen & M.Abbasi	Am1009	Iran: Azarbaiyan-W	LR700271	LR700284	LR700299	LR700256	6
A. obliquum L.	O-6	Russia: BG Ekaterinenburg	HG794228	n.d.	n.d.	n.d.	6

A. obliquum	O-37	Russia: Bashkortostan, Kraka Range	HG794230	n.d.	n.d.	n.d.	1
A. ochroleucum W. & K.*	Am540	Slovenia: Caven	LR700272	LR700285	LR700300	LR700257	6
*A. ochroleucum**	Am542	Slovenia: Nanos	LR700273	LR700286	LR700301	LR700258	6
A. palentinum Losa & P.Monts.	MA532503	Spain: Velilla del Rio Carrion	OM030259	OM032828	OM032836	OM055645	t.s.
A. palentinum	MA778505	Spain: Palencia, ladera N del Espigüete	OM030260	OM032829	OM032837	OM055646	t.s.
A. palentinum	MA515202	Spain: Pico Espigüete, Velilla del Río Carrión	OM030261	OM032830	OM032838	n.d.	t.s.
A. palentinum	SALA136402	Spain: León, Maraña, valle de Valverde	OM030262	OM032831	OM032839	n.d.	t.s.
A. *platyspathum* Schrenk	Tax2905	Kazakhstan: Almaty, Bolschaja Almaatinka	AJ411878	MW201150	n.d.	MW201101	6
A. *platyspathum*	Am1104	Kazakhstan: Toksanbai Range	MW208988	MW201151	n.d.	MW201102	6
A. *platyspathum* subsp. *amblyophyllum* (Kar. & Kir.) N.Friesen	Am903	Kazakhstan: Kungei Alatoo, Fluss Kurmerty	MW208990	MW201152	n.d.	MW201103	6
A. *platyspathum* subsp. *amblyophyllum*	Am1103	Kazakhstan: Kajandytau, Tamshi valey	MW208992	MW201153	n.d.	MW201104	6
A. rubriflorum (Adamovic) Anackov, N.Friesen & Seregin	GL-87	Serbia: East Serbia, Niš, Sićevo Gorge	HG794196	n.d.	n.d.	n.d.	1
A. rubriflorum	GL-88	Serbia: East Serbia, Babušnica, Koritnićka Gorge	HG794197	n.d.	n.d.	n.d.	1
A. rude J.M.Xu	Hexj0446	n.d.	HQ690270	n.d.	n.d.	n.d.	12
A. rude	n.d.	n.d.	MH066485	n.d.	n.d.	n.d.	3
A. rude	n.d.	China	n.d.	MH992112	MH992112	MH992112	5

A. strictum Schrad.	n.d.	China: Xinjiang, Tuoli	GU566621	n.d.	n.d.	n.d.	4
A. strictum	Tax 5404	Kazakhstan: Chu-Ili Mts., Pass Kurdai	AJ411952**	n.d.	n.d.	n.d.	13
A. suaveolens Jacq.	Am888	Germany: Bavaria	LR700274	LR700287	LR700302	LR700259	6
A. suaveolens	Am935	Slovenia: Ankaran	LR700275	LR700288	LR700303	LR700260	6
A. xichuanense J.M.Xu	Am977	China: Nanjiang	LR700276	LR700289	LR700304	LR700261	6
A. xichuanense	1001i	n.d.	MH383264	n.d.	n.d.	n.d.	3
A. xinlongense e D.F. Xie & X.J. He	n.d.	China: BanMaXian, QingHai	MN866565	n.d.	n.d.	n.d.	10
A. xinlongense	n.d.	China: DaoFuXian, SiChuan	MN866573	n.d.	n.d.	n.d.	10

Ref., reference; **n.d.**, no datum; **t.s.**, this study; *, species reported in the article as *A. ericetorum* Thore; **, incorrect GenBank accession in the literature reference.

References

1. Seregin, A.P.; Anackov G.; Friesen, N. 2015. Molecular and morphological revision of the *Allium saxatile* group (Amaryllidaceae): geographical isolation as the driving force of underestimated speciation. *Botanical Journal of the Linnean Society*, **2015**, *178*, 67-101, doi:10.1111/boj.12269
2. Friesen, N.; Vesselova, P.; Osmonaly, B.; Sitpayeva, G.; Luferov, A.; Shmakov, A. *Allium toksanbaicum* (Amaryllidaceae), a new species from Southeast Kazakhstan. *Phytotaxa*, **2021**, *494*, 251-267, doi: 10.11646/phytotaxa.494.3.1
3. GenBank database, submission by Yu, X.H. **2018**.
4. Li, Q.Q.; Zhou, S.D.; He, X.J.; Yu, Y.; Zhang, Y.C.; Wei, X.Q. Phylogeny and biogeography of *Allium* (Amaryllidaceae: Allieae) based on nuclear ribosomal internal transcribed spacer and chloroplast *rps*16 sequences, focusing on the inclusion of species endemic to China. *Annals of Botany*, **2010**, *106*, 709-733, doi:10.1093/aob/mcq177
5. GenBank database, submission by Xie, D.F. **2019**.
6. Friesen, N.; Abbasi, M.; Murtazaliev, R.; Fritsch, R.M. *Allium matinae*—a new species from northwestern Iran. *Phytotaxa*, **2020**, *433*, 181-189, doi:10.11646/phytotaxa.433.3.1
7. GenBank database, submission by Xie, C.; Zhong, Y.; Guo, X.L.; He, X.J. **2018**.
8. Wu, L.L.; Cui, X.K.; Milne, R.I.; SUN, Y.S.; Liu, J.Q. Multiple autopolyploidizations and range expansion of *Allium przewalskianum* Regel. (Alliaceae) in the Qinghai-Tibetan Plateau. *Molecular Ecology*, **2010**, *19*, 1691-1704, doi: 10.1111/j.1365-294X.2010.04613.x

9. Yang, X.; Xie, D.F.; Chen, J.P.; Zhou, S.D.; Yu, Y.; He, X.J. Comparative analysis of the complete chloroplast genomes in *Allium* Subgenus *Cyathophora* (Amaryllidaceae): phylogenetic relationship and adaptive evolution. BioMed Research International, **2020**, *2020*, doi:10.1155/2020/1732586
10. Li, M.J.; Yu, H.X.; Guo, X.L.; He, X.J. Out of the Qinghai–Tibetan Plateau and rapid radiation across Eurasia for *Allium* section *Daghestanica* (Amaryllidaceae). *AoB Plants*, **2021**, *13*, plab017, doi: 10.1093/aobpla/plab017
11. Fritsch, R.M.; Friesen, N. *Allium oreotadzhikorum* and *Allium vallivanchense*, two new species of *Allium* subg. *Polyprason* (Alliaceae) from the Central Asian republic Tajikistan. *Feddes Repertorium*, **2009**, *120*, 3-4, 221-231, doi:10.1002/fedr.200911199
12. Li, D.Z.; Gao, L.M.; Li, H.T.; Wang, H.; Ge, X.J.; Liu, J.Q.; et al. From the cover: comparative analysis of a large dataset indicates that internal transcribed spacer (ITS) should be incorporated into the core barcode for seed plants. *Proceedings of the National Academy of Science of the United States of America*, **2011**, *108*, 19641-19646, doi:10.1073/pnas.1104551108
13. Friesen, N.; Fritsch, R.M.; Blattner, F.R. Phylogeny and new infrageneric classification of *Allium* L. (Alliaceae) based on nuclear ribosomal DNA ITS sequences. *Aliso*, **2006**, *22*, 372–395, doi:10.5642/aliso.20062201.31
